# Sfrp5 increases glucose-stimulated insulin secretion in the rat pancreatic beta cell line INS-1E

**DOI:** 10.1371/journal.pone.0213650

**Published:** 2019-03-28

**Authors:** Maren Carstensen-Kirberg, Karin Röhrig, Corinna Niersmann, D. Margriet Ouwens, Bengt F. Belgardt, Michael Roden, Christian Herder

**Affiliations:** 1 Institute for Clinical Diabetology, German Diabetes Center, Leibniz Center for Diabetes Research at Heinrich Heine University Düsseldorf, Düsseldorf, Germany; 2 German Center for Diabetes Research (DZD), Partner Düsseldorf, Düsseldorf, Germany; 3 Institute for Clinical Biochemistry and Pathobiochemistry, German Diabetes Center, Leibniz Center for Diabetes Research at Heinrich Heine University Düsseldorf, Düsseldorf, Germany; 4 Department of Endocrinology, Ghent University Hospital, Ghent, Belgium; 5 Institute for Beta Cell Biology, German Diabetes Center, Leibniz Center for Diabetes Research at Heinrich Heine University Düsseldorf, Düsseldorf, Germany; 6 Division of Endocrinology and Diabetology, Medical Faculty, Heinrich Heine University, Düsseldorf, Germany; 7 Medical Faculty, Heinrich Heine University, Düsseldorf, Germany; Medical University of Vienna, AUSTRIA

## Abstract

Previous studies reported that secreted frizzled-related protein-5 (Sfrp5) decreases beta cell proliferation and increases fasting insulin levels, but studies on direct effects of Sfrp5 on insulin secretion and its underlying mechanisms are missing. This study examined effects of Sfrp5 on (i) beta cell viability and proliferation, (ii) basal and glucose-stimulated insulin secretion and (iii) canonical and non-canonical Wnt signalling pathways. We incubated rat INS-1E cells with 0.1, 1 or 5 μg/ml recombinant Sfrp5 for 24h. We measured basal and glucose-stimulated insulin secretion at glucose concentrations of 2.5 and 20 mmol/l. Phosphorylated and total protein content as well as mRNA levels of markers of cell proliferation, canonical and non-canonical Wnt signalling pathways were examined using Western blotting and real-time PCR. Differences between treatments were analysed by repeated measurement one-way ANOVA or Friedman’s test followed by correction for multiple testing using the Benjamini-Hochberg procedure. At 5 μg/ml, Sfrp5 reduced mRNA levels of cyclin-B1 by 25% (p<0.05). At 1 and 5 μg/ml, Sfrp5 increased glucose-stimulated insulin secretion by 24% and by 34% (both p<0.05), respectively, but had no impact on basal insulin secretion. Sfrp5 reduced the phosphorylation of the splicing forms p46 and p54 of JNK by 39% (p<0.01) and 49% (p<0.05), respectively. In conclusion, Sfrp5 reduced markers of cell proliferation, but increased in parallel dose-dependently glucose-stimulated insulin secretion in INS-1E cells. This effect is likely mediated by reduced JNK activity, an important component of the non-canonical Wnt signalling pathway.

## Introduction

The secreted frizzled-related protein (Sfrp)5 belongs to the Sfrp family, the largest group of WNT inhibitors [1]. Sfrp5 is a secreted protein which is produced by several human tissues such as visceral and subcutaneous adipose tissue, liver, mononuclear blood cells and pancreatic islets [2–5]. It was found to bind and antagonise Wnt5a, Wnt5b and Wnt11 and therefore to regulate both the canonical and non-canonical Wnt signalling pathway [6]. In murine adipose tissue, Sfrp5 inhibited the non-canonical but not the canonical Wnt signalling pathway [2], whereas Sfrp5 blocked the canonical Wnt signalling pathway in rat beta cells [4]. The impact of Sfrp5 on the non-canonical Wnt signalling pathway has not been investigated in these cells.

Two epidemiological studies investigated the association between Sfrp5 and markers for insulin secretion. We did not find any correlation between circulating Sfrp5 and the homeostasis model assessment for β-cell function (HOMA-B) [7] and this was supported by another human study [8]. On the cellular level, Sfrp5 is downregulated in pancreatic islets of obese rodents and humans [4]. The administration of Sfrp5 reduced the proliferation in primary islet cells [4] and the overexpression of Sfrp5 led to higher serum fasting insulin levels in mice [9].

Currently, no data are available regarding direct effects of Sfrp5 on insulin secretion and the potential underlying mechanism in beta cells in vitro. Therefore, this study aimed to investigate the impact of Sfrp5 on (i) cell viability and proliferation, (ii) basal and glucose-stimulated insulin secretion and (iii) the canonical and non-canonical Wnt signalling pathway in beta cells.

## Material and methods

### Cell culture

We seeded 40,000 INS-1E (AddexBio, San Diego, CA, USA) per cm^2^ (flasks) and cultivated these cells in medium containing RPMI 1640 with glutamine (Life Technologies, Carlsbad, CA, USA), 1 mmol/l sodium pyruvate (Life Technologies), 50 μmol/l β-mercaptoethanol (Life Technologies), 10 mmol/l HEPES (Life Technologies), 10% fetal bovine serum (FBS) (Biochrom, Berlin, Germany), 100 IU/ml penicillin and 100 μg/ml streptomycin (Life Technologies). After 4 days, INS-1E were detached using 0.05% trypsin (Life Technologies), seeded at 100,000 cells per cm^2^ (24- or 96-well plate) and cultured in the aforementioned medium for another 3 days. Then, cells were fasted for FBS for 4h and treated with 0.1, 1 or 5 μg/ml recombinant Sfrp5 (R&D Systems, Wiesbaden, Germany) for 24h. INS-1E cells were also incubated with 0.2 ng/ml interleukin (IL)-1β (R&D Systems) as positive control for the inhibition of cell viability. Treatment of the cells with 10 μmol/l CHIR99021 (Miltenyi Biotec GmbH, Bergisch Gladbach, Germany) served as positive control of an activated canonical Wnt signalling pathway.

### Cell viability test

We used the CellTiter-Glo luminescent cell viability assay (Promega, Madison, WI, USA) according to the manufacturer´s instructions to determine the number of viable cells in culture. This test is based on the quantitation of the ATP content in the cells which is an indicator of metabolically active cells. Ribonucleotide adenosintriphosphates (rATPs) (Promega) were used for the standard curve. In addition, we used a colorimetric cell viability kit I (CCVK-I, WST-8) (PromoCell, Heidelberg, Germany) to assess potential cytotoxic effects of Sfrp5 on INS-1E cells.

### Basal and glucose-stimulated insulin secretion

We incubated cells first in glucose-free medium (RPMI 1640 with glutamine, w/o glucose; Life Technologies) for 2h at 37°C, then in warm glucose-free K/H buffer for 30 min at 37°C followed by 30 min in prewarmed K/H buffer containing 2.5 or 20 mmol/l glucose. Thereafter, we immediately collected the supernatant, centrifuged it for 15 sec. at 8000*g at room temperature and kept 400 μl of the supernatant in a new tube. We added 500 μl of lysis buffer (1% Triton X-100, 20 mmol/l HEPES pH 7.9, 0.3 mmol/l NaCl, 1.5 mmol/l MgCl_2_, 0.2 mmol/l EDTA, cOmplete Mini Protease Inhibitor Cocktail and PhosStop (both from Roche, Mannheim, Germany)) per well and kept the plate on ice for 30 min. Then, we collected the lysed cells, centrifuged them for 15 sec. at 8000*g at room temperature and kept 200 μl of the lysed cells in a new tube. Supernatant and cell lysate were stored at -80°C. We measured the insulin concentration in supernatant and lysate using the ultra-sensitive rat insulin ELISA Kit from Crystal Chem (Elk Grove Village, IL, USA).

### Western blotting

For the extraction of whole protein lysate of the INS-1E cells, we used RIPA buffer containing 30 mmol/l Tris-HCl (pH 7.5), 1 mmol/l EDTA, 150 mmol/l NaCl, 1% TritonX-100, 1% sodium-deoxycholate, 1% NP-40, cOmplete Mini Protease Inhibitor Cocktail and PhosStop. Samples were sonicated at 50% amplitude for 30 seconds with pulses for 5s following breaks for 2s while samples were kept on ice. Then samples were centrifuged at 14,000*g for 20 minutes at 4°C. The supernatant was removed and frozen at -80°C. We analysed protein abundance and phosphorylation by Simple Western size-based assays [10,11] using a 12–230 kDa Separation Module (ProteinSimple, San Jose, CA, USA) according to the manufacturer´s instructions. A Simple Western is an automated Western without gels, transfer devices, blots, films and manual analysis. The chemiluminescence is measured at different exposure times and automatically quantified by Compass for Simple Western software (version 3.1.7) (ProteinSimple). The chemiluminescent signal is shown as an electropherogram. Examples for electropherograms are shown in S1 Fig. The software calculates the area under the curve (AUC) of the peak to generate a virtual blot-like image [12]. The primary antibodies for non-phospho (active) beta-catenin, phospho-cAMP response element-binding protein (Creb) (Ser133), phospho-protein kinase C (PKC) (pan) (ßII Ser 660) and phospho-stress-activated protein kinase/c-Jun N-terminal kinase SAPK/JNK (Thr183/Tyr185) (81E11) were purchased from Cell Signaling Technology (Danvers, MA, USA). We used anti-rabbit and anti-mouse secondary antibodies, respectively (both from ProteinSimple). All signals were normalised for equal loading with antibodies for α-tubulin (Cell Signaling Technology).

### RNA isolation and real-time PCR

RNA was isolated from the INS-1E cells and real-time PCR was performed as described before [13]. We measured mRNA levels of cyclin-B1 (CCNB1), Ki-67 (MKI67), proliferating-cell-nuclear-antigen (PCNA), transcription factor 7-like 2 (TCF7L2), c-Myc (MYC) and cyclin-D1 (CCND1) and normalised to mRNA levels of eukaryotic initiation factor 4A-II (EIF4A2) as reference gene using the QuantiTect Primer Assay (QIAGEN, Hilden, Germany).

### Statistical analysis

Differences between treatments were analysed by repeated measurement one-way (analysis of variance) ANOVA or Friedman’s test (depending on data distribution in the control group) followed by the Benjamini-Hochberg method as correction for multiple testing. All data are presented as mean + SEM. Statistical analyses were performed using the Prism 7 (GraphPad Software, La Jolla, CA, USA) software. P-values <0.05 were considered as statistically significant.

## Results

### Sfrp5, cell viability and proliferation

The treatment of INS-1E cells with 0.1, 1 or 5 μg/ml Sfrp5 for 24h had no impact on the ATP content of these cells as marker for cell viability compared to untreated cells (S2 Fig, S1 Data).

**Fig 1:**
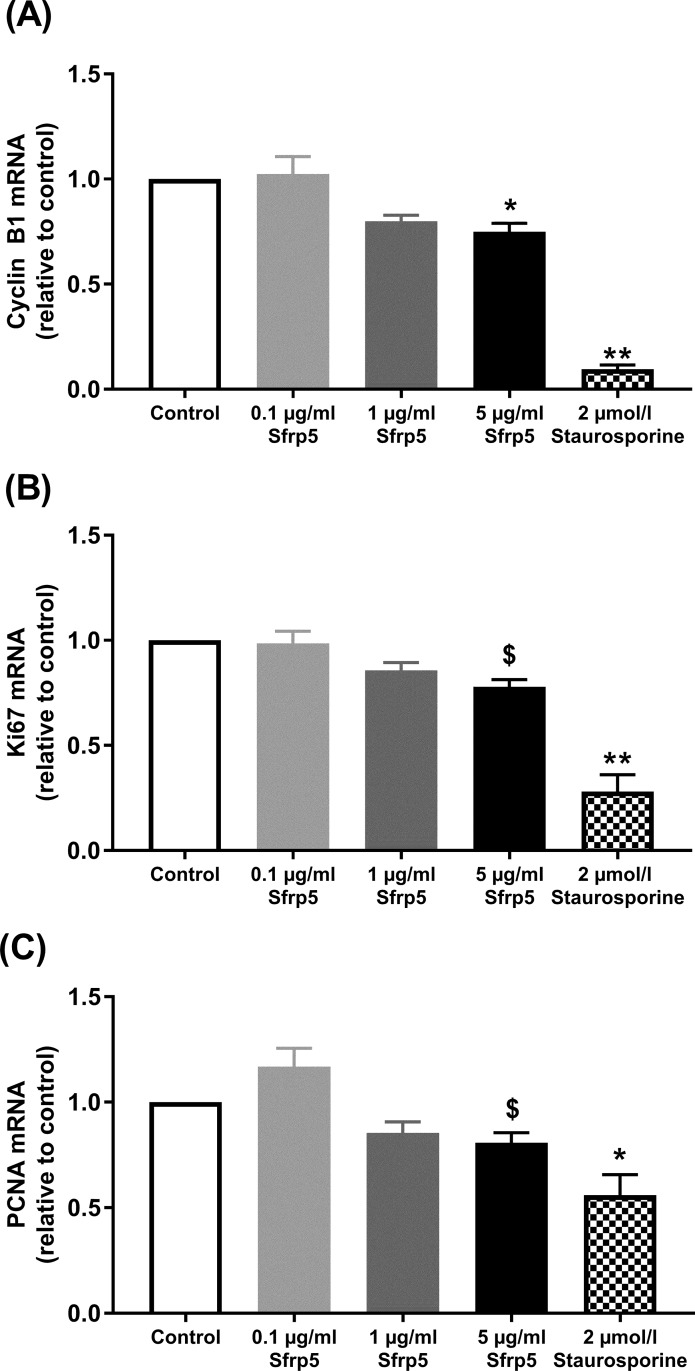
Sfrp5 and cell proliferation. Effect of different concentrations of Sfrp5 on markers of proliferation: (A) Cyclin-B1 (N = 5), (B) Ki67 (N = 5) and (C) proliferating-cell-nuclear-antigen (PCNA) (N = 5) in INS-1E cells. Data were normalised to eukaryotic initiation factor 4A-II (EIF4A2). Staurosporine was used as positive control for inhibition of cell proliferation. Data are presented as mean + SEM (fold change relative to control). $, p = 0.056; *, p<0.05; **, p<0.01 vs. control.

However, 5 μg/ml Sfrp5 reduced mRNA levels of markers of cell proliferation. Cyclin-B1 mRNA was decreased by 25% (p<0.05), whereas we observed only a trend towards lower mRNA levels of Ki67 (-22%) and PCNA (-19%) (both p = 0.056). The treatment of INS-1E with 0.1 or 1 μg/ml did not affect mRNA levels of cyclin-B1, Ki67 and PCNA (Fig 1, S1 Data). We also used a colorimetric cell viability kit I (WST-8) to test for potential cytotoxic effects of Sfrp5 on INS-1E cells. We did not find any differences between untreated INS-1E cells and those treated with the different concentrations of Sfrp5.

### Sfrp5 and insulin secretion

**Fig 2:**
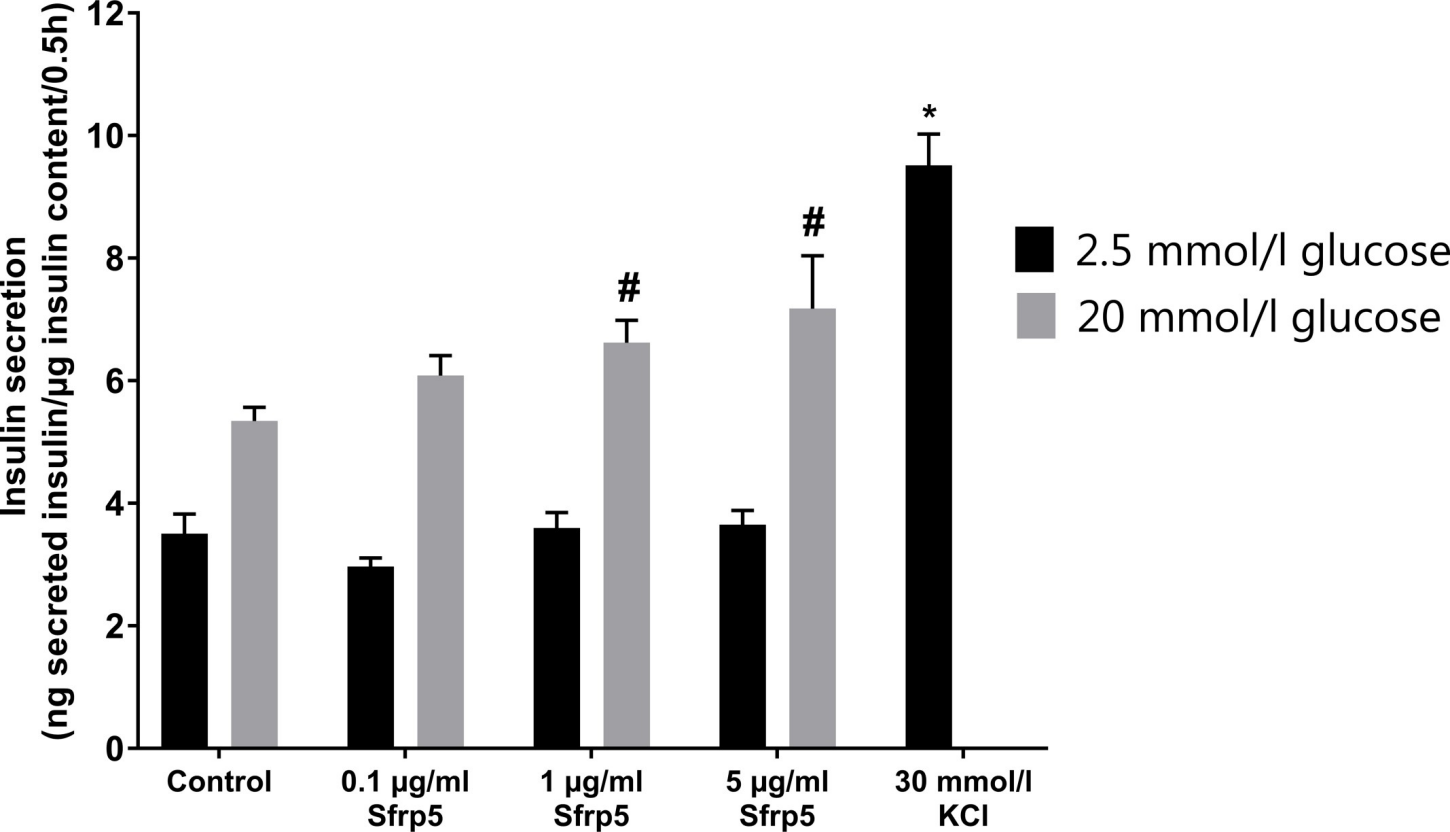
Sfrp5 and insulin secretion. Effect of different concentrations of Sfrp5 on basal and glucose-stimulated insulin secretion of INS-1E cells (N = 5). KCl was the positive control for increased insulin secretion. Data are presented as mean + SEM. #, p<0.05 vs. control (20 mmol/l glucose); ***, p<0.001 vs. control (2.5 mmol/l glucose).

Sfrp5 dose-dependently increased glucose-stimulated insulin secretion by 24% by 1 μg/ml (p<0.05) and 34% by 5 μg/ml (p<0.05) in INS-1E cells. The lowest dose of Sfrp5 had no significant impact on glucose-stimulated insulin secretion. The administration of 0.1, 1 or 5 μg/ml Sfrp5 for 24h had no impact on basal insulin secretion of INS-1E cells. KCl, serving as positive control, increased basal insulin secretion by 172% (p<0.001) (Fig 2, S1 Data).

### Sfrp5 and the Wnt-signalling pathway

**Fig 3:**
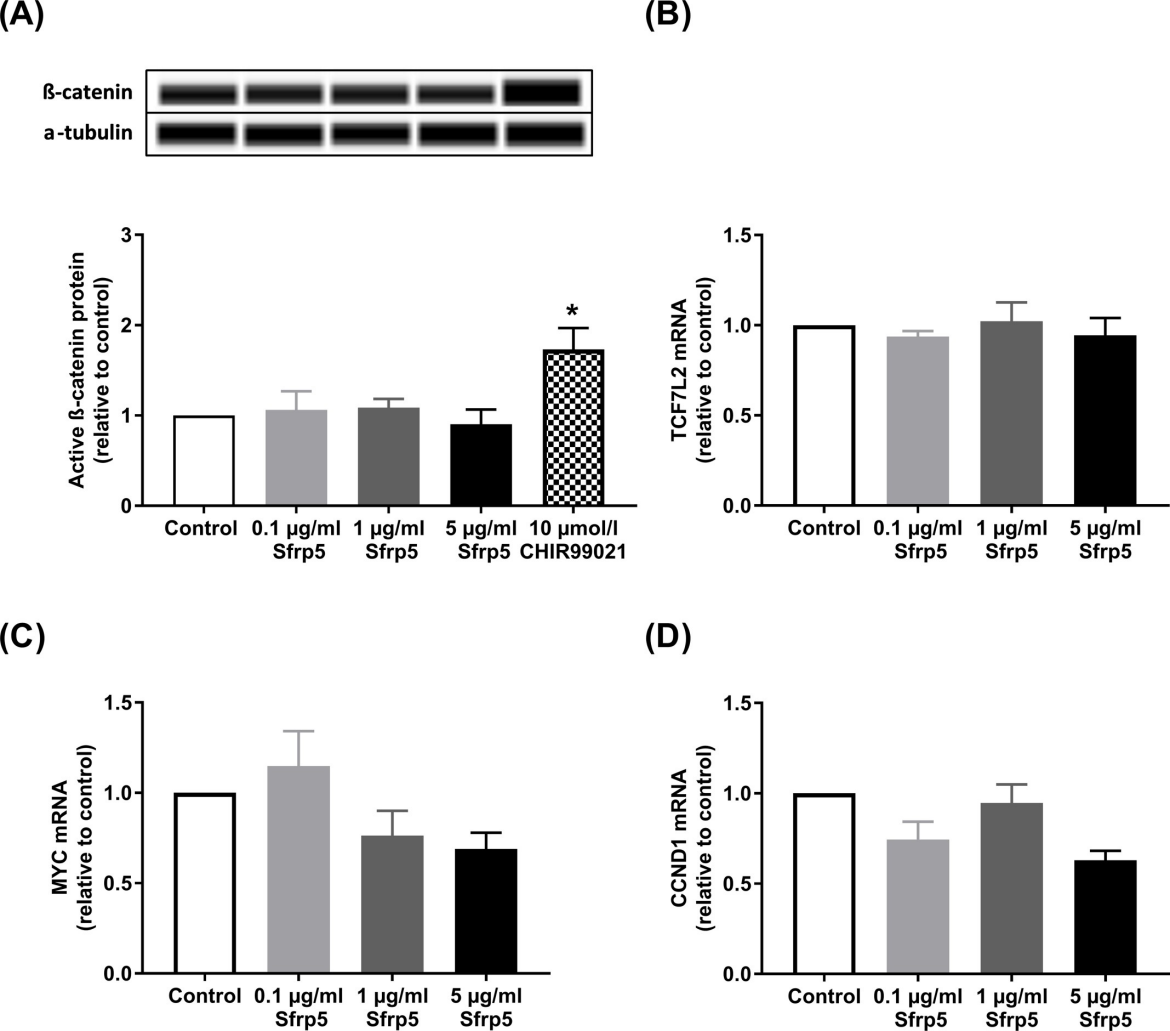
Sfrp5 and canonical Wnt signalling. Effect of different concentrations of Sfrp5 on markers and target genes of the canonical Wnt-signalling pathway: (A) protein levels of the active form of the non-phospho β-catenin (92 kDa) (N = 5), (B) mRNA levels of the transcription factor 7-like 2 (TCF7L2) (N = 4), (C) mRNA levels of MYC (N = 4) and (D) mRNA levels of CCND1 (N = 4) (normalised to eukaryotic initiation factor 4A-II (EIF4A2)) in INS-1E cells. The loading control for the Western blot was α-tubulin (52 kDa). CHIR99021 is a positive regulator of the canonical Wnt-signalling pathway. Data are presented as mean + SEM (fold change relative to control). *, p<0.05 vs. control.

None of the tested concentrations of Sfrp5 affected the protein abundance of the active form of β-catenin (Fig 3A, S1 Data). CHIR99021 is a positive regulator of the canonical Wnt-signalling pathway and increased the protein levels of β-catenin by 73% (p<0.05). Sfrp5 also did not affect mRNA levels of the transcription factor TCF7L2, c-Myc and cyclin-D1 (Fig 3B–3D, S1 Data).

**Fig 4:**
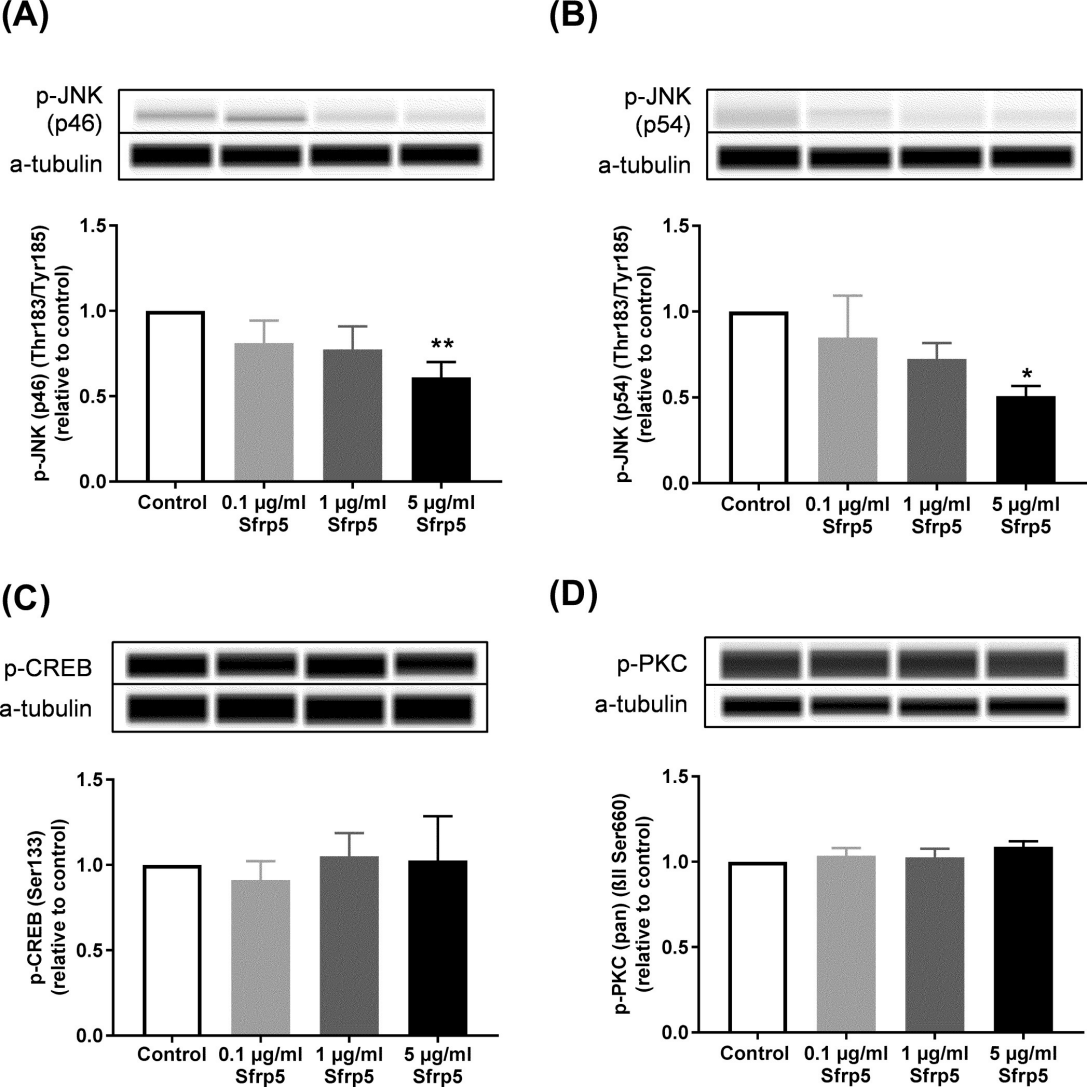
Sfrp5 and non-canonical Wnt signalling pathway. Effect of different concentrations of Sfrp5 on phosphorylation levels of markers of the non-canonical Wnt-signalling pathway: (A) c-Jun N-terminal kinases (JNK) (p46) (46 kDa) (N = 5), (B) JNK (p54) (54 kDa) (N = 5) (C) cAMP response element-binding protein (CREB) (43 kDa) (N = 5) and (D) protein kinase C (PKC) α, β I, β II, δ, ε, η and θ isoforms (78, 80, 82 and 85 kDa) (N = 5) in INS-1E cells. The loading control was α-tubulin (52 kDa). Data are presented as mean + SEM (fold change relative to control). *, p<0.05; **, p<0.01 vs. control.

Treatment of INS-1E cells with 5 μg/ml Sfrp5 decreased the phosphorylation levels of the two splicing forms of JNK p46 by 39% (p<0.01) and p54 by 49% (p<0.001), respectively, at threonine-183 and tyrosine-185, but had no impact on serine-133 phosphorylation of CREB at and serine-660 phosphorylation of PKC (α, β I, β II, δ, ε, η and θ isoforms). Both 0.1 and 1 μg/ml Sfrp5 did not affect the phosphorylation of the isoforms of JNK, CREB and PKC (Fig 4, S1 Data).

## Discussion

The main findings of this study were that Sfrp5 (i) had no impact on cell viability but reduced markers of cell proliferation, (ii) dose-dependently increased glucose-stimulated but not basal insulin secretion and (iii) reduced JNK activity, an important target of the non-canonical Wnt signalling pathway in INS-1E cells.

### Sfrp5, cell viability and proliferation

In our study, Sfrp5 had no impact on the viability of INS-1E cells as measured by intracellular ATP content. However, we found that Sfrp5 reduced markers of cell proliferation in these cells. Our data are in line with previous in vitro studies. One study reported that Sfrp5 decreased the proliferation rate in primary rat islets, whereas Sfrp5 knockdown increased the proliferation in primary rat islets and INS-1E cells [4]. Another study found that the overexpression of Sfrp5 reduced proliferation of INS-1 cells and dispersed rat islets only under high (16.7 mmol/l) but not under basal glucose concentrations (5.6 mmol/l) [14]. Our cell culture medium for INS-1E cells contained 11.1 mmol/l glucose indicating that this glucose concentration is high enough for Sfrp5 to regulate the proliferation of INS-1E cells.

### Sfrp5 and insulin secretion

Sfrp5 dose-dependently increased glucose-stimulated, but not basal insulin secretion in INS-1E. Our findings are in contrast to currently available epidemiological studies. We did not find any association between serum Sfrp5 and HOMA-B in overweight individuals [7]. This was confirmed by another human study in overweight individuals and patients with type 2 diabetes or prediabetes [8]. However, these studies were based on small samples and used HOMA-B as surrogate marker of beta-cell function, which is calculated from fasting levels of glucose and insulin and thereby does not necessarily reflect the postprandial situation. An in vitro study found that the overexpression of Sfrp5 increased fasting serum insulin levels in lean and obese mice [9]. Unfortunately, it is unclear which Sfrp5 concentration was achieved by Sfrp5 overexpression and glucose-stimulated insulin secretion was not measured, which precludes a direct comparison with our findings.

In the present study, 5 μg/ml Sfrp5 reduced markers of cell proliferation, but increased glucose-stimulated insulin secretion in INS-1E cells. A decrease of beta cell mass has been associated with reduced glucose-stimulated insulin secretion. However, several studies found that beta cell function is more relevant than beta cell mass in this context and beta cells are able to compensate for the insulin demand by increased insulin secretion despite reduced beta cell number [15]. At present, further studies are necessary to explore whether INS-1E cells compensate reduced cell number by increased insulin secretion after prolonged Sfrp5 treatment.

Our study indicates that Sfrp5 might have a protective role in beta cell function in INS-1E. This is in line with results from the population-based Cooperative Health Research in the Region of Augsburg (KORA) F4 study including 1161 participants. Here, higher Sfrp5 was inversely associated with multiple risk factors for type 2 diabetes and cardiovascular diseases as well as lower odds of prediabetes/type 2 diabetes suggesting a protective role of Sfrp5 in the development of cardiometabolic diseases [16].

### Sfrp5 and Wnt signalling pathway

We found that Sfrp5 reduced the activity of JNK in INS-1E cells. JNK is an important component of the non-canonical Wnt signalling pathway [17] and has been described to be regulated by Sfrp5 in different murine cell types which is in line with our findings from rat beta cells. Obese mice overexpressing Sfrp5 had reduced phosphorylation levels of JNK in fat tissues compared to control mice [2] and Sfrp5 reversed Wnt5a stimulated JNK phosphorylation in macrophages [18] So far, there is only one study showing that a cytokine (tumour necrosis factor α) inhibited glucose-stimulated insulin secretion through activation of JNK in INS-1 cells [19] indicating that reduced JNK activity in our experimental setting might be involved in the increased insulin secretion induced by Sfrp5. However, we cannot exclude that Sfrp5 also affects factors/processes that are directly involved in the insulin secretion procedure such as calcium concentration, K+ channels, insulin expression, vesicle formation etc.

Sfrp5 had no impact on β-catenin and TCF7L2 as downstream targets as well as c-Myc and cyclin-D1 as target genes of the canonical Wnt signalling pathway in INS-1E. Another study showed that reduced Sfrp5 levels for 48h led to increased levels of β-catenin and TCF7L2 in INS-1E cells suggesting that Sfrp5 may block the canonical Wnt signalling pathway [4]. However, further studies examining the effects of higher Sfrp5 levels are not available, so that the dose-response relationship between Sfrp5 treatment and the activation of the canonical Wnt signalling pathway merits further investigations.

As INS-1E cells are a radiation-induced rat beta cell line from insulinoma, we are currently not able to transfer these results to primary beta cells. Further studies are necessary to investigate the impact of Sfrp5 on insulin secretion in isolated islets and/or primary beta cells from rats and humans and to evaluate whether Sfrp5 might be of therapeutic interest for the treatment of obesity and diabetes.

## Conclusion

In INS-1E cells, Sfrp5 decreased markers of cell proliferation, but dose-dependently stimulated glucose-stimulated insulin secretion. The Sfrp5-induced insulin secretion may be mediated by reduced JNK activity indicating involvement of the non-canonical, but not the canonical Wnt signalling pathway.

## Supporting information

S1 Fig Examples of Simple Western electropherograms. Peaks of β-catenin and α-tubulin of untreated samples (A), samples treated with 0.1 μg/ml Sfrp5 (B), 1 μg/ml Sfrp5 (C), 5 μg/ml Sfrp5 (D) or 10 μmol/l CHIR99021 (E). The compass software calculated the area under the curve (AUC) of the peaks and generated bands in virtual blot-like images.
(PDF)

S2 Fig Sfrp5 and cellular ATP content. Effect of different concentrations of Sfrp5 on the viability of INS-1E cells as assessed by the ATP content in the cells using a luminescence assay (N = 5). IL-1β served as marker of inhibition of ATP production. Data are presented as mean + SEM. ***, p<0.001 vs. control.
(TIF)

S1 Data The tables show all raw data which support the corresponding figures. (XLSX)
