# Supplementary figures and images for: Sfrp5 increases glucose-stimulated insulin secretion in the rat pancreatic beta cell line INS-1E
Source: PLoS One. 2019 Mar 28;14(3):e0213650. doi: 10.1371/journal.pone.0213650 (PMC6438539; doi:10.1371/journal.pone.0213650)

**(A)**

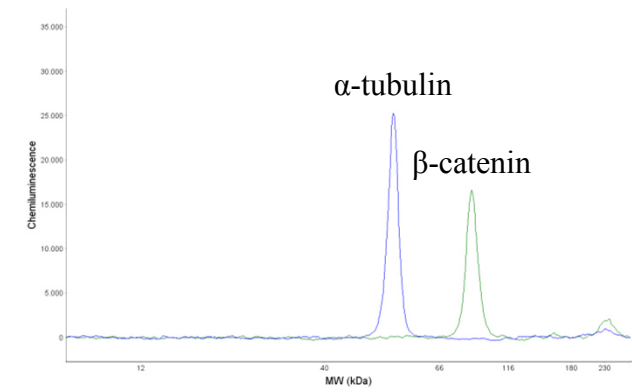

**(B)**

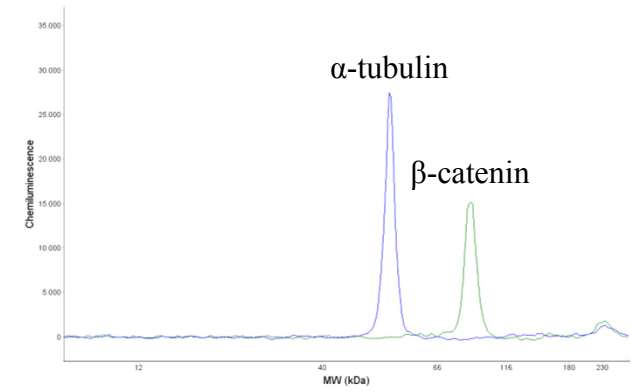

**(C)**

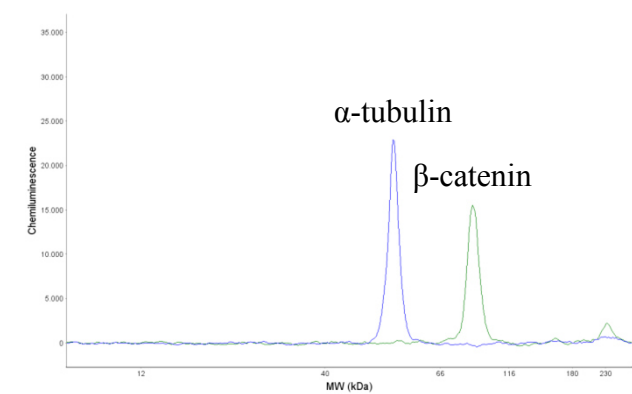

**(D)**

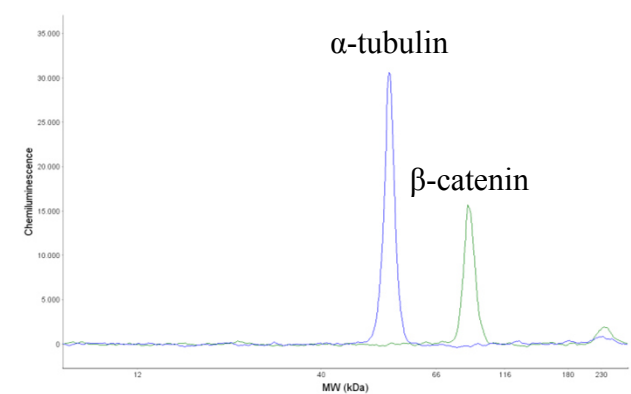

**(E)**

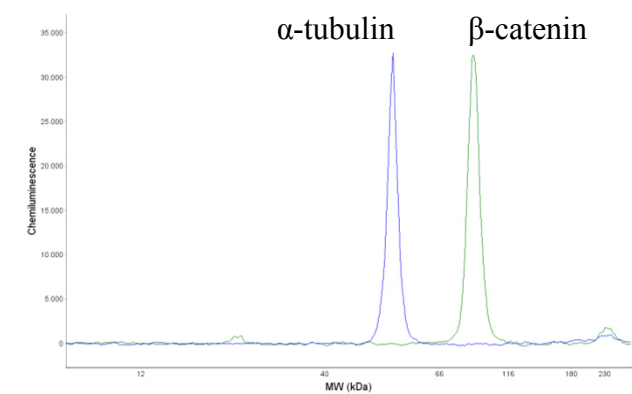

Supplement: S1 Fig — Peaks of β-catenin and α-tubulin of untreated samples (A), samples treated with 0.1 μg/ml Sfrp5 (B), 1 μg/ml Sfrp5 (C), 5 μg/ml Sfrp5 (D) or 10 μmol/l CHIR99021 (E). The compass software calculated the area under the curve (AUC) of the peaks and generated bands in virtual blot-like images. (PDF) [file pone.0213650.s001.pdf]
